# Síndrome Vasoplégica sob Oxigenação por Membrana Extracorpórea: Tratamento Bem-Sucedido com Azul de Metileno

**DOI:** 10.36660/abc.20250070

**Published:** 2025-06-18

**Authors:** Ahmet Aydin, Banu Katlan, Selman Kesici

**Affiliations:** 1 Hacettepe University Department of Cardiovascular Surgery Ankara Turquia Hacettepe University - Department of Cardiovascular Surgery, Ankara – Turquia; 2 Hacettepe University Department of Pediatric Critical Care Medicine Ankara Turquia Hacettepe University - Department of Pediatric Critical Care Medicine, Ankara – Turquia

**Keywords:** Vasoplegia, Oxigenação por Membrana Extracorpórea, Azul de Metileno

## Abstract

A síndrome vasoplégica é um estado de hipotensão que se desenvolve devido à baixa resistência vascular sistêmica e está associada a uma alta taxa de mortalidade. O azul de metileno é conhecido por melhorar a hipotensão e reduzir os requisitos de vasopressores ao aumentar a resistência na vasoplegia pela inibição da óxido nítrico sintase. Aqui, pretendemos relatar o uso e a eficácia do azul de metileno para tratar cinco casos de síndrome vasoplégica gravemente hipotensivos, apesar da oxigenação por membrana extracorpórea. Concluímos que o azul de metileno pode ser usado como um tratamento salva-vidas para a síndrome vasoplégica refratária aos tratamentos de suporte padrão, incluindo a oxigenação por membrana extracorpórea.

## Introdução

A síndrome vasoplégica (SV) é uma hipotensão arterial grave com alta mortalidade. As características mais características são baixa resistência vascular sistêmica (RVS) e débito cardíaco (DC) normal ou alto. A redução da RVS é o principal mecanismo fisiopatológico,^1–4^ Uma resposta inflamatória sistêmica (RIS), caracterizada por hiperpolarização celular, deficiência relativa de vasopressina com aumento dos níveis de óxido nítrico (NO) induzível, desempenha um papel na diminuição da RVS. Os fatores de risco conhecidos são transfusão de sangue, circulação extracorpórea (CEC), trauma, transplante, queimadura, sepse e uso de medicamentos específicos, como inibidores da enzima conversora de angiotensina, bloqueadores dos canais de cálcio (CCB), antagonista da angiotensina II, heparina, amiodarona, aprotinina e protamina.^3–5^ Volume intravenoso e vasopressores, azul de metileno (AM) e hidroxocobalamina em altas doses são usados no tratamento.^5,6^ Sabe-se que aumenta a RVS pela inibição da NO sintase e, consequentemente, melhora a hipotensão no SV e reduz as necessidades de vasopressores.^7–10^ No entanto, há informações limitadas disponíveis sobre o tratamento da hipotensão refratária em pacientes submetidos à oxigenação por membrana extracorpórea (ECMO). Recentemente, tem havido um interesse crescente no uso de AM para o tratamento do SV que se desenvolve durante a ECMO. Neste contexto, apresentamos casos em que AM foi empregado para tratar efetivamente pacientes gravemente hipotensos, apesar do suporte de ECMO na presença de SV.

## Série de casos

Este estudo inclui dados de pacientes entre os anos de 2004 e 2013 que receberam AM devido a SV enquanto estavam em suporte de ECMO, e está em conformidade com a discussão sobre as diretrizes e normas regulatórias de pesquisa envolvendo seres humanos; Conselho Nacional de Saúde, Ministério da Saúde, Brasília, DF. Resolução 466/2012. (Hacettepe Comitê de Ética do Hospital Universitário, Número de Aprovação: 2019/25-28 data: 22/10/2019). Todos os participantes forneceram consentimento informado.

O AM foi administrado com Bluemet 100 mg/10 ml (Vem İlaç, Istambul, Turquia).

Os dados incluíram idade, sexo, peso, diagnóstico primário, fluxo de ECMO, duração, sinais vitais, escore de inotrópico vasoativo (EIV) antes e depois do tratamento com AM, parâmetros laboratoriais (gases sanguíneos e funções orgânicas) e resultado.

O EIV é calculado da seguinte forma:


dopamina(mcg/kg/min)+dobutamina(mcg/kg/min)+100×epinefrina(mcg/kg/min)+10×milrinona(mcg/kg/min)+100×norepinefrina(mcg/kg/min)+10.000×vasopressina(U/kg/min).

Cinco pacientes foram incluídos no estudo. A idade média foi de 45,8 ± 29,2 anos (13-91 anos). Três (60%) pacientes eram do sexo masculino. O diagnóstico primário incluiu doença cardíaca coronária (n: 1), doença valvar cardíaca grave (n: 2), infecção por COVID-19 (n: 1) e intoxicação por CCB (n: 1). A indicação de ECMO foi baixo DC em todos os pacientes. A duração mediana do suporte de ECMO foi de 2,6 ± 1,8 dias (1-6 dias). Três pacientes foram desmamados do suporte de ECMO e 2 pacientes morreram. As características demográficas dos pacientes são mostradas na Tabela 1.

**Tabela 1:** Características do paciente e resultados da ECMO com azul de metileno

Paciente n°.		1	2	3	4	5
Características dos pacientes						
**Idade/Gênero**		62/M	47/F	13 /F	16 /M	91/ M
**Peso**		70 kg	62 kg	45 kg	70 kg	81 kg
**Diagnóstico Primer**		Doença cardíaca congênita	RM grave	MIS-C	Intoxicação por CCBs.	EA crítica
**Indicação de ECMO**		LCOS pós-operatório	LCOS pós-operatório	LCOS	LCOS	LCOS pós-operatório
**Parâmetros ECMO**						
	Taxa de fluxo	5,7 L/minuto	5,5 L/minuto	4,5 L/minuto	5,5 L/minuto	6 L/minuto
	RPM	2500	3000	2500	2500	3000
	Fio2	80	90	80	95	95
	Anticoagulação	20 U/kg/h	20 U/kg/h	20 U/kg/h	20 U/kg/h	20 U/kg/h
**Azul de metileno**						
	Dose	2 mg/kg	1 mg/kg	2 mg/kg	1 mg/kg	1 mg/kg
	Tempo	Dose única	Dose única	1 dose	Dose única	2 doses
	Efeitos colaterais	Nenhum	Nenhum	Nenhum	Nenhum	Nenhum
**Escore Inotrópico Vasoativo**						
	Antes do AM	50	40	50	50	80
	30 min de infusão de AM	30	20	40	30	80
	60 min de infusão de AM	20	10	30	20	80
	30 min após a infusão de AM	20	5	25	10	80
	60 min após infusão de AM	10	5	15	5	80
	4 horas após a infusão de AM	10	5	5	0	-
	8 horas após a infusão de AM	10	5	5	0	-
	12 horas após a infusão de AM	5	0	5	0	-
	24 horas após a infusão de AM	0	0	5	0	-
	2 dias após infusão de AM	0	0	5	0	-
**Duração da ECMO**		2 dias	1 dia	6 dias	3 dias	4 horas
**Resultado**		Descarregado	Descarregado	Descarregado	Morte cerebral	Saída

ECMO: oxigenação por membrana extracorpórea; AM: azul de metileno; CHD: doença cardíaca coronária; MR: regurgitação mitral; MIS-C: síndrome inflamatória multissistêmica em crianças; CCBs: bloqueadores de canais de cálcio; EA: estenose aórtica; RPM: revoluções por minuto.

**Figura 1:**
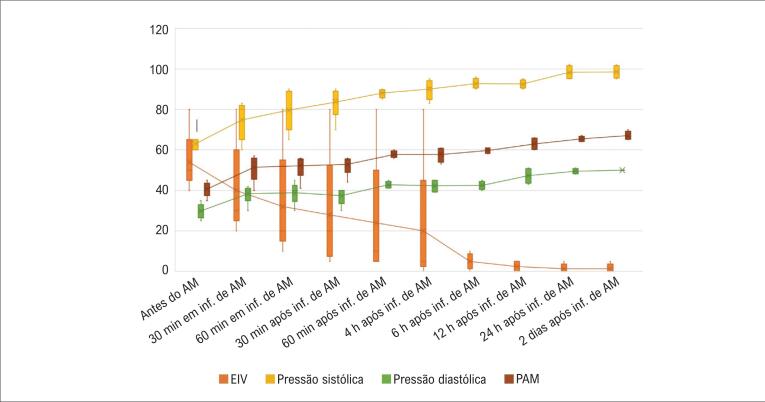
Pressão arterial e evolução do EIV dos pacientes após tratamento com AM. (EIV: Escore de Inotrópico Vasoativo; AM: Azul de Metileno; PA: Pressão Arterial; PAM: Pressão Arterial Média; Antes do AM: Antes do Início da Infusão de Azul de Metileno; 30 min em AM inf: 30 minutos em infusão de azul de metileno; 60 min em AM inf: 60 minutos em infusão de azul de metileno; 30 min após AM inf: 30 minutos após infusão de azul de metileno; 60 min após AM inf: 60 minutos após infusão de azul de metileno; 4 h após AM inf: 4 horas após infusão de azul de metileno; 8 h após AM inf: 8 horas após infusão de azul de metileno; 12 h após AM inf: 12 horas após infusão de azul de metileno; 24 h após AM inf: 24 horas após infusão de azul de metileno; 2 dias após AM inf: 2 dias após infusão de azul de metileno). (Barra horizontal: "mediana" do escore do inotrópico vasoativo e valores de pressão arterial; Xs; as caixas, ou seja, intervalos interquartis são os percentis 25 e 75 da pontuação do inotrópico vasoativo e valores de pressão arterial; Extremo superior do whisker: o maior escore do inotrópico vasoativo e pressão arterial (90° percentil), excluindo valores discrepantes; Extremo inferior do whisker: o menor escore do inotrópico vasoativo e pressão arterial (10° percentil), excluindo valores discrepantes; Pontos: os maiores e menores valores discrepantes do escore do inotrópico vasoativo e pressão arterial).

Imediatamente após o diagnóstico de SV ser considerado, AM foi iniciado em uma dose de 1-2 mg/kg/dose por infusão por 1 hora. Uma resposta rápida foi obtida em 4 pacientes. Nos primeiros 60 minutos de tratamento, a pressão arterial pôde ser medida, a pressão arterial média foi >60 mm Hg, e a retirada do tratamento com inotrópicos e vasopressores foi iniciada. Antes de AM ser administrado, o EIV médio foi 50 sob ECMO e diminuiu para 20 na metade do tratamento com AM. Os pacientes não precisaram de vasopressores e inotrópicos na 12ª hora. Dois pacientes foram desmamados da ECMO em 24 horas. O paciente com RIS foi acompanhado em ECMO por 6 dias sem a necessidade de tratamentos com inotrópicos e vasopressores após AM e foi desmamado com sucesso. O paciente com overdose de CCB deu uma resposta completa ao tratamento com AM, mas desenvolveu morte cerebral em 72 horas. A resposta parcial ao tratamento com AM foi obtida em nosso paciente de 91 anos que foi submetido a cirurgia de revascularização do miocárdio e AVR. A pressão arterial foi medida no final da infusão de AM, mas morreu 6 horas depois devido à instabilidade hemodinâmica. O curso da pressão arterial e EIV dos pacientes antes e depois de AM é mostrado na Figura 1.

## Discussão

A SV é uma condição caracterizada por uma pressão arterial média (PAM) baixa, DC normal ou alto e SVR baixa, não responsiva a vasopressores em altas doses. A RIS caracterizada por hiperpolarização celular, uma deficiência relativa de vasopressina com um aumento no NO induzível e os níveis de c-GMP desempenham um papel na diminuição da SVR.^11^ A inibição da superprodução e atividade de NO e c-GMP desempenha um papel crítico no tratamento da vasoplegia refratária.^6,11^ No presente estudo, em 5 casos com SV, três pacientes foram pós-cardiotomia, um paciente teve intoxicação por CCB e um paciente teve RIS (Tabela 1). Independentemente da etiologia, foi relatado que a taxa de mortalidade chega a 50% em pacientes com VS.^12^ Há dados mínimos disponíveis sobre o SV em pacientes submetidos à ECMO. Mesmo que a indicação de ECMO do paciente não seja uma das condições relacionadas ao SV mencionadas acima, o próprio conjunto de ECMO pode causar inflamação e levar ao SV. No tratamento, catecolaminas são usadas para aumentar a RVS. As opções de tratamento, além das catecolaminas, são limitadas.^13^

Considerando os mecanismos fisiopatológicos subjacentes à vasoplegia refratária, a inibição da superprodução e atividade de NO e cGMP torna-se crucial. Portanto, o AM é usado tanto em experimentos com animais quanto em humanos no tratamento, o que neutraliza o aumento da estimulação de NOS ao antagonizar a atividade endotelial de NOS e inibir a atividade da guanilato ciclase.^7,8,14^ O uso de AM para hipotensão foi relatado pela primeira vez em 1999 em um paciente com dificuldade de desmame da CEC.^15^ Hoje, o AM é usado em crianças e adultos no tratamento de choque séptico, anafilaxia, CEC e cirurgia, e vasoplegia relacionada à intoxicação por medicamentos, e resultados promissores são relatados. A terapia com AM em SV reduz a necessidade de suporte inotrópico, possivelmente devido à atenuação da lesão de isquemia/reperfusão, além de uma redução nos requisitos de vasopressores. Além disso, estudos relataram que a pressão arterial média e a RVS aumentaram, a frequência cardíaca diminuiu e o CO e a resistência vascular pulmonar não mudaram em pacientes que receberam AM.^16^

SV pode ocorrer comumente em pacientes submetidos a CEC. Vários fatores contribuem, incluindo hemodiluição aguda, administração de citrato, RIS e aumento da atividade de NO. Estudos existentes destacaram a aplicação bem-sucedida de AM antes, durante ou após CEC para prevenir e/ou tratar VS.^17^ Foi relatado que um paciente com vasoplegia grave, não responsivo ao tratamento convencional após cirurgia cardíaca, apresentou parâmetros hemodinâmicos normalizados após receber um único bolus intravenoso de 2 mg/kg de AM. Em outro caso envolvendo um paciente com transplante cardíaco, AM foi efetivamente usado para restaurar a estabilidade hemodinâmica no pós-operatório quando a infusão de norepinefrina não produziu os resultados desejados. Da mesma forma, SVR e PAM mais altos, requisitos reduzidos de vasopressores e uma menor incidência de SV no grupo AM foram documentados em um estudo comparativo que investigou o efeito hemodinâmico da administração de AM no início do CPB.^18^

O uso de AM foi relatado recentemente no tratamento de SV sob ECMO.^19^ Ortoleva et al.^20^ investigaram o uso de AM em 45 pacientes de ECMO com SV, relatando uma resposta positiva em mais de 50% dos pacientes, com uma redução na dosagem de norepinefrina observada dentro de uma a duas horas após a administração de AM. Além disso, uma tendência para melhor sobrevivência até a alta foi observada.

Relatamos 5 casos que desenvolveram SV resistente a vasopressores e inotrópicos, apesar de ECMO ter recebido AM para tratamento. Nossos pacientes adultos foram pós-cardiotomia. Os diagnósticos de nossos outros dois pacientes adolescentes foram envenenamento por CCB e RIS associada à COVID-19. Nesses pacientes cujo DC foi garantido com ECMO, um diagnóstico de SV foi concluído. Eles estavam recebendo infusão de NA em altas doses e infusão de adrenalina e dopamina em altas doses. Como nossos casos, foi relatado na série de casos que a resposta hemodinâmica ao AM começou a ser obtida nas primeiras horas.

Gillman^21^ destacou potenciais efeitos colaterais, incluindo metemoglobinemia, hemólise, náusea, vômito, dor abdominal, dor no peito, edema pulmonar, arritmia cardíaca, toxicidade do sistema nervoso central, interferem com oxímetros de pulso, especialmente em uma dose > 4 mg / kg. Não observamos nenhum efeito colateral em nossos pacientes, exceto a coloração da urina em azul, que é o efeito colateral mais comum. O efeito do AM por meio da inibição do NO pode ser importante em termos de desencadear hipertensão pulmonar. No entanto, doses terapêuticas não afetam a RVP. Da mesma forma, os achados clínicos de hipertensão pulmonar não se desenvolveram em nossos pacientes. Portanto, a avaliação cuidadosa do uso do AM é crucial.

## Conclusão

AM tem sido usado como um tratamento salva-vidas para SV, apesar dos tratamentos de suporte padrão. SV pode se desenvolver apesar do suporte de ECMO, e AM deve ser mantido em mente em tais casos. Até onde sabemos, os casos atuais são os primeiros casos de uso efetivo de AM em ECMO para SV. A opinião de que AM é uma bala mágica no tratamento de SV enfatizada em publicações anteriores foi confirmada por nossos pacientes.

## Disponibilidade de Dados

Os conteúdos subjacentes ao texto da pesquisa estão contidos no manuscrito.
