# Supplementary material for: Síndrome Vasoplégica sob Oxigenação por Membrana Extracorpórea: Tratamento Bem-Sucedido com Azul de Metileno
Source: Arq Bras Cardiol. 2025 Jun 18;122(5):e20250070. [Article in Portuguese] doi: 10.36660/abc.20250070 (PMC12212369; doi:10.36660/abc.20250070)
Supplement: Translation

# Translation: Vasoplegic Syndrome under Extracorporeal Membrane Oxygenation: Successful Treatment with Methylene Blue

**DOI:** 10.36660/abc.20250070i

**Authors:** Ahmet Aydin, Banu Katlan, Selman Kesici

**Affiliations:** 1 Ankara Türkiye Hacettepe University - Department of Cardiovascular Surgery, Ankara – Türkiye; 2 Ankara Türkiye Hacettepe University - Department of Pediatric Critical Care Medicine, Ankara – Türkiye

**Keywords:** Vasoplegia, Extracorporeal Membrane Oxygenation, Methylene Blue

## Abstract

A vasoplegic syndrome is a state of hypotension that develops due to low systemic vascular resistance and is associated with a high mortality rate. Methylene blue is known to improve hypotension and reduce vasopressor requirements by increasing resistance in vasoplegia by inhibiting nitric oxide synthase. Here, we aimed to report on the use and effectiveness of methylene blue to treat severely hypotensive five vasoplegic syndrome cases despite extracorporeal membrane oxygenation. We concluded that Methylene blue can be used as a lifesaving treatment for vasoplegic syndrome refractory to standard supportive treatments, including ECMO.

## Introduction

Vasoplegic syndrome (VS) is a serious arterial hypotension with high mortality. The most characteristic features are low systemic vascular resistance (SVR) and normal or high cardiac output (CO). The reduction of SVR is the main pathophysiological mechanism.^1–4^ A systemic inflammatory response (SIRS), characterized by cellular hyperpolarization, a relative vasopressin deficiency with an increase in inducible nitric oxide (NO) levels, plays a role in decreasing SVR. Known risk factors are blood transfusion, cardiopulmonary bypass (CPB), trauma, transplantation, burn, sepsis, and use of specific drugs such as angiotensin-converting enzyme inhibitors, calcium-channel blockers (CCBs), angiotensin-II antagonist, heparin, amiodarone, aprotinin and protamine.^3–5^ Iv volume and vasopressors, methylene blue (MB), and high-dose hydroxocobalamin are used in the treatment.^5,6^ It is known to increase SVR by inhibiting NO synthase and consequently improve hypotension in VS and reduce vasopressor requirements.^7–10^ However, limited information is available on the management of refractory hypotension in patients undergoing extracorporeal membrane oxygenation (ECMO). Recently, there has been growing interest in the using of MB for the management of VS that develops during ECMO. In this context, we present cases in which MB was employed to effectively treat severely hypotensive patients despite ECMO support in the presence of VS.

## Case series

This study includes data of patients between the years 2004 and 2013 who received MB due to VS while undergoing ECMO support, and It complies with discussion on the guidelines and regulatory standards of research involving human beings; National Health Council, Ministry of Health, Brasilia, DF. Resolution 466/2012. (Hacettepe University Hospital Ethical Committee, Approval Number: 2019/25-28 date: 22/10/2019). All participants provided informed consent.

Given MB was Bluemet 100 mg/10 ml (Vem İlaç, İstanbul, Turkey).

The data included age, gender, weight, primary diagnosis, ECMO flow, duration, vital signs, vasoactive inotrope score (VIS) before and after MB treatment, laboratory parameters (blood gases and organ functions), and outcome.

VIS is calculated as follows:


dopamine(mcg/kg/min)+dobutamine(mcg/kg/min)+100×epinephrine(mcg/kg/min)+10×milrinone(mcg/kg/min)+100×norepinephrine(mcg/kg/min)+10.000×vasopressin(U/kg/min).

Five patients were included in the study. The mean age was 45.8±29.2 years (13-91 years). Three (60%) patients were male. Primary diagnosis included coronary heart disease (n: 1), severe heart valve disease (n:2), COVID-19 infection (n:1), and CCB intoxication (n:1). The ECMO indication was low CO in all patients. The median duration of ECMO support was 2.6 ±1.8 days (1-6 days). Three patients were weaned from ECMO support, and 2 patients died. Demographic characteristics of patients are shown in Table 1.

**Table 1:** Patient Characteristics and ECMO Outcomes with Methylene Blue

Patient No.		1	2	3	4	5
Patients Characteristics						
**Age/ Gender**		62/M	47/F	13 /F	16 /M	91/ M
**Weight**		70 kg	62 kg	45 kg	70 kg	81 kg
**Primer Diagnosis**		Postop	Severe MR	MIS-C	CCBs intox.	Critical AR
**ECMO Indication**		LCOSCHD	Postop LCOS	LCOS	LCOS	Postop LCOS
**ECMO Parametres**						
	Flow Rate	5,7 L/minute	5,5 L/minute	4,5 L/minute	5,5 L/minute	6 L/minute
	RPM	2500	3000	2500	2500	3000
	Fio2	80	90	80	95	95
	Anticoagulation	20 U/kg/h	20 U/kg/h	20 U/kg/h	20 U/kg/h	20 U/kg/h
**Methylene blue**						
	Dose	2 mg/kg	1 mg/kg	2 mg/kg	1 mg/kg	1 mg/kg
	Time	Single dose	Single dose	1 doses	Single dose	2 doses
	Side effects	None	None	None	None	None
**Vasoactive Inotrop Score**						
	Before MB	50	40	50	50	80
	30 min into MB infusion	30	20	40	30	80
	60 min into MB infusion	20	10	30	20	80
	30 min after MB infusion	20	5	25	10	80
	60 min after MB infusion	10	5	15	5	80
	4 hrs after MB infusion	10	5	5	0	-
	8 hrs after MB infusion	10	5	5	0	-
	12 hrs after MB infusion	5	0	5	0	-
	24 hrs after MB infusion	0	0	5	0	-
	2 days after MB infusion	0	0	5	0	-
**ECMO duration**		2 days	1 day	6 days	3 days	4 hrs
**Outcome**		Discharged	Discharged	Discharged	Brain death	Exitus

ECMO: extracorporeal membrane oxygenation; MB: methylene blue; CHD: coronary heart disease; MR: mitral regurgitation; MIS-C: multi-system ınflammatory syndrome in children; CCBs: Ca channel blockers; AR: aort stenosis; RPM: revolutions per minute.

Immediately after diagnosis of VS was considered, MB was initiated at a dose of 1-2 mg/kg/dose by infusion for 1 hour. A rapid response was obtained in 4 patients. Within the first 60 minutes of treatment, blood pressure could be measured, mean arterial pressure was >60 mm Hg, and withdrawal from inotrope and vasopressor treatment was initiated. Before MB was given, the mean VIS was 50 under ECMO and decreased to 20 in half of the MB treatment. The patients did not need vasopressor and inotropes at the 12th hour. Two patients were weaned from ECMO within 24 hours. The patient with SIRS was followed up at ECMO for 6 days without the need for inotropes and vasopressor treatments after MB and was successfully weaned. The patient with CCB overdose gave a complete response to MB treatment but developed brain death at 72 hours. Partial response to MB treatment was obtained in our 91-year-old patient who underwent CABG and AVR. Blood pressure was measured at the end of MB infusion, but died 6 hours later due to hemodynamic instability. The course of patients’ blood pressure and VIS before and after MB is shown in Figure 1.

**Figure 1:** Patients’ blood pressure and VIS course following MB treatment. (VIS: Vasoactive Inotrop Score; MB: Methylene blue; BP: Blood pressure; MAP: Mean arterial pressure; Before MB: Before Methylene Blue Infusion Initiation; 30 min into MB inf: 30 minutes into methylene blue infusion; 60 min into MB inf: 60 minutes into methylene blue infusion; 30 min after MB inf: 30 minutes after methylene blue infusion; 60 min after MB inf: 60 minutes after methylene blue infusion; 4 hrs after MB inf: 4 hours after methylene blue infusion; 8 hrs after MB inf: 8 hours after methylene blue infusion; 12 hrs after MB inf: 12 hours after methylene blue infusion; 24 hrs after MB inf: 24 hours after methylene blue infusion; 2 days after MB inf: 2 days after methylene blue infusion). (Horizontal bar: "median" vasoactive inotrope score and blood pressure values; Xs; the boxes, i.e., interquartile intervals are 25th and 75th percentile vasoactive inotrope score and blood pressure values; Upper extreme of whisker: the highest vasoactive inotrope score and blood pressure (90th percentile), excluding outliers; Lower extreme of whisker: the lowest vasoactive inotrope score and blood pressure (10th percentile), excluding outliers; Dots: the largest and smallest outliers vasoactive inotrope score and blood pressure values).

## Discussion

VS is a condition characterized by a low MAP, normal or high CO, and low SVR unresponsive to high-dose vasopressors. SIRS characterized by cellular hyperpolarization, a relative vasopressin deficiency with an increase in inducible NO, and c-GMP levels play a role in decreasing SVR.^11^ Inhibition of both NO and c-GMP overproduction and activity plays a critical role in the treatment of refractory vasoplegia.^6,11^ In the present study, in 5 cases with VS, three patients were post-cardiotomy, one patient had CCB intoxication, and one patient had SIRS (Table 1). Regardless of the etiology, it has been reported that the mortality rate reaches 50% in patients with VS.^12^ There is minimal data available regarding the VS in patients undergoing ECMO. Even if the ECMO indication of the patient is not one of the above-mentioned VS-related conditions, the ECMO set itself may cause inflammation and lead to VS. In treatment, catecholamines are used to increase SVR. Treatment options, in addition to catecholamines, are limited.^13^

Considering pathophysiological mechanisms underlying refractory vasoplegia, the inhibition of both NO and cGMP overproduction and activity becomes crucial. Therefore, MB is used in both animal experiments and humans in the treatment, which counteracts increased NOS stimulation by antagonizing endothelial NOS activity and inhibiting guanylate cyclase activity.^7,8,14^ The use of MB for hypotension was first reported in 1999 in a patient with difficulty in weaning from CPB.^15^ Today, MB is used in both children and adults in the treatment of septic shock, anaphylaxis, CPB and surgery, and drug intoxication-related vasoplegia, and promising results are reported. MB therapy in VS reduces the need for inotropic support, possibly due to attenuation of ischemia/reperfusion injury, in addition to a reduction in vasopressor requirements. Moreover, studies have reported that mean arterial pressure and SVR increased, heart rate decreased, and CO and pulmonary vascular resistance did not change in patients given MB.^16^

VS may commonly occur in patients undergoing CPB. Various factors contribute, including acute hemodilution, citrate administration, SIRS, and increased NO activity. Existing studies have highlighted the successful application of MB before, during, or after CPB to prevent and/or treat VS.^17^ It was reported that a patient with severe vasoplegia, unresponsive to conventional treatment after cardiac surgery, experienced normalized hemodynamic parameters after receiving a single 2 mg/kg intravenous bolus of MB. In another case involving a heart transplant patient, MB was effectively used to restore hemodynamic stability postoperatively when the infusion of norepinephrine did not yield the desired results. Similarly, higher SVR and MAP, reduced vasopressor requirements, and a lower incidence of VS in the MB group were documented in a comparative study investigating the hemodynamic effect of MB administration at the initiation of CPB.^18^

The use of MB has recently been reported in the treatment of VS under ECMO.^19^ Ortelova et al.^20^ investigated the use of MB in 45 ECMO patients with VS, reporting a positive response in over 50% of patients, with a reduction in norepinephrine dosage observed within one to two hours after MB administration. Additionally, a trend towards improved survival to discharge was noted.

We reported 5 cases who developed VS resistant to vasopressors and inotropes despite ECMO given MB for treatment. Our adult patients were post-cardiotomy. The diagnoses of our other two adolescent patients were CCB poisoning and COVID-19-associated SIRS. In these patients whose CO was guaranteed with ECMO, a diagnosis of VS was concluded. They were receiving high-dose NA infusion and high-dose adrenaline and dopamine infusion. Like our cases, it has been reported in the case series that hemodynamic response to MB started to be obtained within the first hours.

Gillman^21^ highlighted potential side effects, including methemoglobinemia, hemolysis, nausea, vomiting, abdominal pain, chest pain, pulmonary edema, cardiac arrhythmia, central nervous system toxicity, interfere with pulse oximeters, especially on a dose > 4 mg / kg. We did not see any side effects in our patients except staining the urine color blue, which is the most common side effect. The effect of MB through NO inhibition could be important in terms of triggering pulmonary hypertension. However, therapeutic doses do not affect PVR. Similarly, clinical findings of pulmonary hypertension did not develop in our patients. Therefore, careful evaluation of MB usage is crucial.

## Conclusion

MB has been used as a lifesaving treatment for VS despite the standard supportive treatments. VS may develop despite ECMO support, and MB must be kept in mind in such cases. To our knowledge, the current cases are the first cases of effective use of MB in ECMO for VS. The opinion that MB is a magic bullet in the treatment of VS emphasized in previous publications has been confirmed by our patients.

## Data Availability

The underlying content of the research text is contained within the manuscript.
